# Independent regulation of Piezo1 activity by principal and intercalated cells of the collecting duct

**DOI:** 10.1016/j.jbc.2023.105524

**Published:** 2023-12-01

**Authors:** Kyrylo Pyrshev, Anna Atamanchuk-Stavniichuk, Mariya Kordysh, Oleg Zaika, Viktor N. Tomilin, Oleh Pochynyuk

**Affiliations:** Department of Integrative Biology and Pharmacology, The University of Texas Health Science Center at Houston

**Keywords:** mechanosensitivity, Ca^2+^-signaling, urinary flow, acidosis, Yoda-1

## Abstract

The renal collecting duct is continuously exposed to a wide spectrum of fluid flow rates and osmotic gradients. Expression of a mechanoactivated Piezo1 channel is the most prominent in the collecting duct. However, the status and regulation of Piezo1 in functionally distinct principal and intercalated cells (PCs and ICs) of the collecting duct remain to be determined. We used pharmacological Piezo1 activation to quantify Piezo1-mediated [Ca^2+^]_i_ influx and single-channel activity separately in PCs and ICs of freshly isolated collecting ducts with fluorescence imaging and electrophysiological tools. We also employed a variety of systemic treatments to examine their consequences on Piezo1 function in PCs and ICs. Piezo1 selective agonists, Yoda-1 or Jedi-2, induced a significantly greater Ca^2+^ influx in PCs than in ICs. Using patch clamp analysis, we recorded a Yoda-1-activated nonselective channel with 18.6 ± 0.7 pS conductance on both apical and basolateral membranes. Piezo1 activity in PCs but not ICs was stimulated by short-term diuresis (injections of furosemide) and reduced by antidiuresis (water restriction for 24 h). However, prolonged stimulation of flow by high K^+^ diet decreased Yoda-1-dependent Ca^2+^ influx without changes in Piezo1 levels. Water supplementation with NH_4_Cl to induce metabolic acidosis stimulated Piezo1 activity in ICs but not in PCs. Overall, our results demonstrate functional Piezo1 expression in collecting duct PCs (more) and ICs (less) on both apical and basolateral sides. We also show that acute changes in fluid flow regulate Piezo1-mediated [Ca^2+^]_i_ influx in PCs, whereas channel activity in ICs responds to systemic acid–base stimuli.

Mechanosensitivity is an universal stress-responsive mechanism allowing effective adaption to external physical forces induced by physiological as well as pathological states/conditions (1). Most common cellular responses to mechanical stimuli include cytoskeleton reorganization, activation of various plasma membrane delineated intracellular signaling cascades, and a direct flux of electrolytes *via* mechanoactivated channels (1, 2). Piezo1 was identified as a long-anticipated molecular conduit of the mechanically activated cation currents observed in a variety of cells (3, 4). Functional Piezo1 channel is comprised of three subunits each having 14 transmembrane domains to form a single central cation-conducting pore (5). The overall structure resembles a propeller with three flexible extracellular blades, which are well-suited to transduce mechanical force to the gate of the central pore (5, 6). Patch clamp analysis of Piezo1 in native cells and over-expression systems revealed that application of a negative pressure to the recording pipette (*i.e.* membrane stretch) induces a rapidly inactivating cation-selective current with single channel conductance around 20 pS (4, 7). Further development of channel-selective agonists/activators Yoda-1 (8) and Jedi-2 (9) allowed quantification of the Piezo1-dependent Ca^2+^ influx in different cells without constraints associated with its rapid inactivation. Importantly, it was recently shown that chemical (Yoda-1) and mechanical (hypotonicity) stimuli induce similar actions on Piezo1 blade expansion, which correlate with channel activity (6) indicative of the common underlying mechanism of Piezo1 stimulation.

At the systemic level, Piezo1 was implicated in a variety of essential physiological processes, such as development of vasculature (10, 11), blood pressure control (12), and voiding function (13, 14) to name a few. Piezo1 deletion caused embryonically lethal phenotype in mice due to profound deficiencies in the flow-dependent vascular remodeling during the development (10, 11). On the other hand, gain-of-function Piezo1 mutations have been linked to several clinically relevant syndromes, such as hereditary xerocytosis with fetal hydrops (15, 16). Piezo1 is broadly expressed in both excitable and non-excitable (most commonly epithelial) tissues, including the kidneys (4). However, the physiological significance of Piezo1 in the renal tubule is just beginning to emerge. It was previously shown that conditional Piezo1 deletion does not affect urinary concentrating ability but delays urinary dilution upon rehydration (17). On the other hand, pathological upregulation of Piezo1 levels has been implicated in the progression of renal fibrosis (18). In the renal tubule, Piezo1 expression has been reported predominantly in the late segments from the cortical to inner medullary collecting ducts (17, 19), although a weaker diffuse staining was also detected in glomeruli (19) and proximal tubule (18). The collecting duct is comprised of two cell types: principal and intercalated (PCs and ICs, respectively) exhibiting distinct morphology and physiological roles (20, 21). In particular, more abundant PCs are primarily involved in Na^+^-water reabsorption and K^+^ secretion, while ICs are critical for enacting acid–base transport (20, 21). It is not currently known whether Piezo1 is functional in both cells types and whether it is regulated by the same or cell-type specific mechanisms.

It is well appreciated that the collecting duct is subjected to the highest variations/changes in fluid flow rates and osmotic gradients depending on dietary electrolyte intake and water consumption (22, 23). These mechanical forces are known to induce comparable elevations in [Ca^2+^]_i_ in both PCs and ICs (24, 25), which are instrumental in modulating water-electrolyte transport, for instance, by promoting the flow-induced K^+^ secretion (26). However, published evidence suggests that Piezo1-reporting signal is largely accumulated on the basolateral side (17, 19), which does not align with its role as a flow-sensor. Indeed, we and others have previously shown that the activity of another mechanosensitive [Ca^2+^]_i_ permeable channel, transitransient receptor potential vanilloid channel type 4 (TRPV4) is imperative for flow-dependent [Ca^2+^]_i_ signaling (22) and subsequently flow-induced K^+^ secretion in the collecting duct (27, 28, 29). Interestingly, a functional interplay was shown in different cell types, including acinar cells and chondrocytes, where an initial transient Ca^2+^ influx *via* Piezo1 could lead to sustained TRPV4 activation (30, 31). It is not known whether a similar coupling exists between Piezo1 and TRPV4 in the collecting duct cells.

The current study was undertaken to examine the significance of Piezo1 activation on [Ca^2+^]_i_ signaling in native collecting duct cells. We focused on addressing the following research questions: (1) if Piezo1 can be found on both basolateral and apical membrane? (2) is there a functional interaction between mechanosensitive Piezo1 and TRPV4 in the collecting duct? (3) whether Piezo1 activity is comparable in PCs and ICs? and (4) what systemic stimuli modulate Piezo1 activity in these cell types?

## Results

### Piezo1-dependent [Ca^2+^]_i_ influx is greater in PCs of the collecting duct

**Figure 1:**
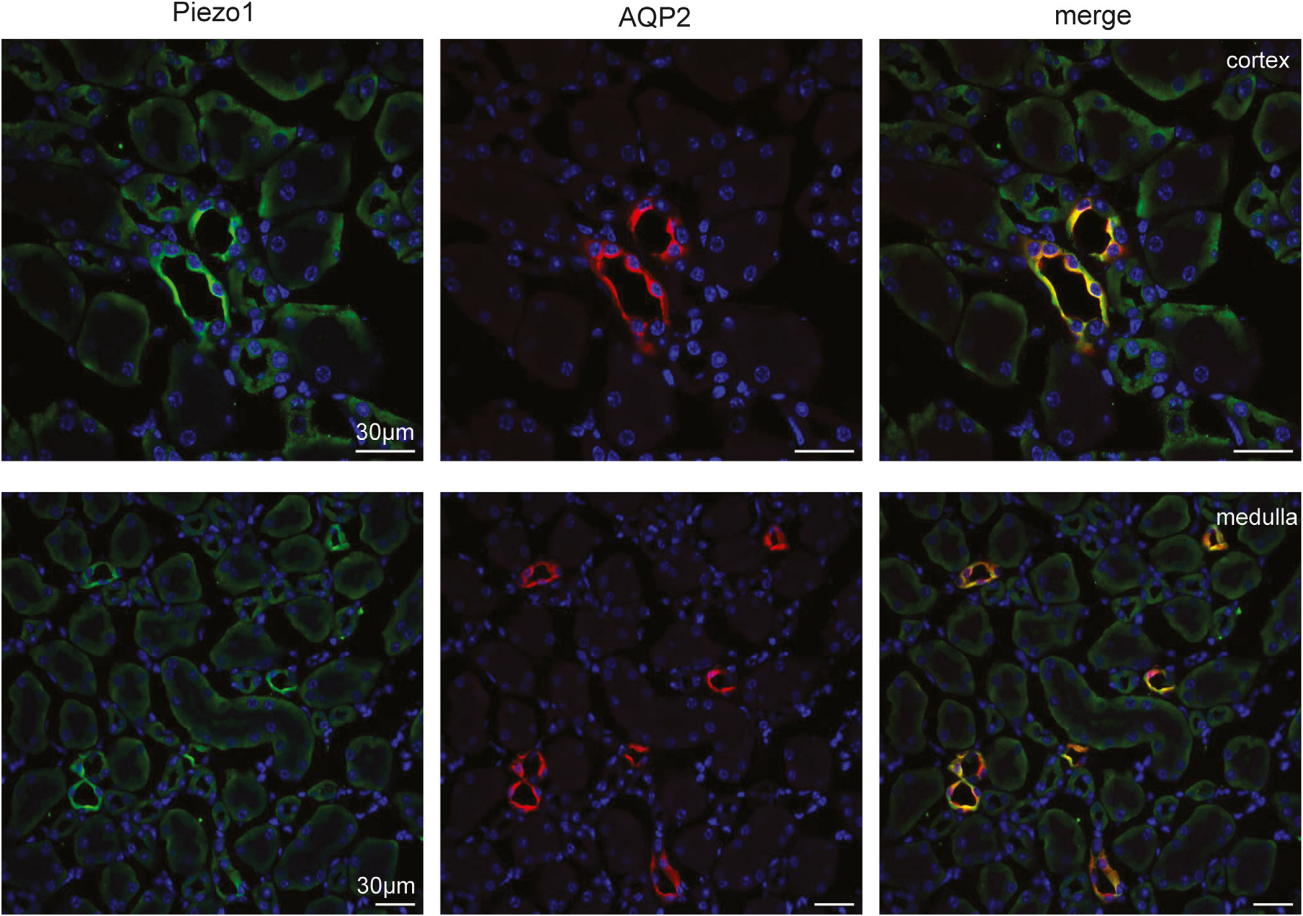
**Piezo1 expression in the kidney is most abundant in the collecting duct.** Representative confocal micrographs showing Piezo1 (*pseudocolor green*), AQP2 (*pseudocolor red*) reporting signals, and the merged image in renal cortical (*top row*) and medullary (*bottom row*) sections. Nuclear DAPI staining is shown with *pseudocolor blue*. Piezo1-reporting signal predominantly localizes to the AQP2-positive (collecting duct) segments of the renal nephron. At the same time, a weaker basolateral Piezo1 signal is present in AQP2-negative renal tubules. AQP2, aquaporine 2; DAPI, 4′,6-diamidino-2-phenylindole.

Previous studies using transgenic reporter mice showed the most abundant Piezo1 expression in the collecting duct (17, 19). In agreement, our immunofluorescence confocal microscopy found the strongest Piezo1 signal in AQP2-positive tubular segments in both cortical and medullary renal sections (Fig. 1). Here, Piezo1 signal was present on both apical and basolateral sides, whereas AQP2-negative tubular segments exhibited much weaker basolateral staining.

**Figure 2:**
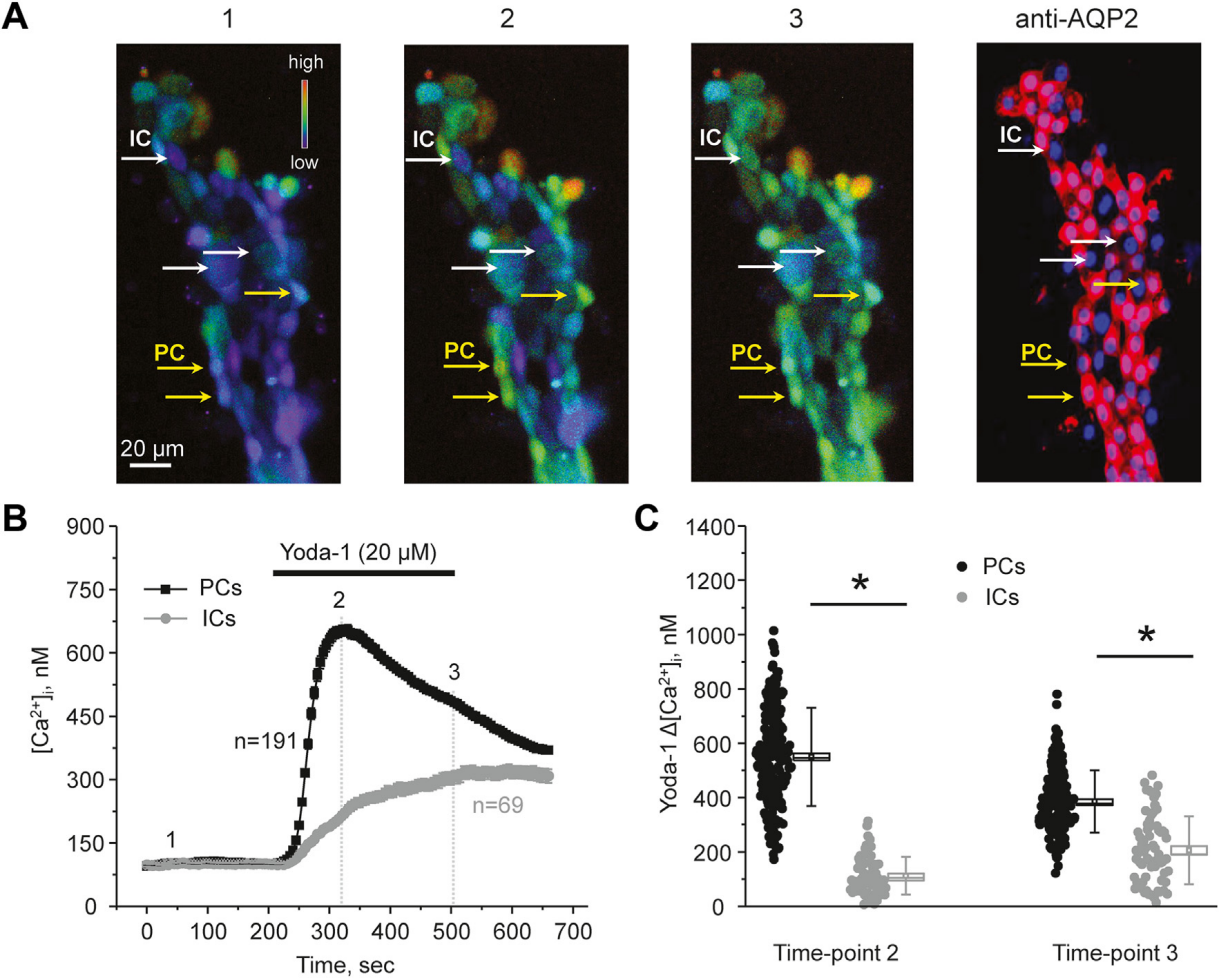
**A selective Piezo1 agonist, Yoda-1 induces different [Ca**^**2+**^**]**_**i**_**responses in principal (PCs) and intercalated (ICs) cells of the collecting duct.***A*, representative *pseudocolor* images (*blue*-low and red-high) of an isolated split-opened collecting duct loaded with Ca^2+^-sensitive dye fura-2 at the baseline (1), after 30 s (2), and 5 min (3) of 20 μM Yoda-1 application. Shown on the right is confocal micrograph of the same split-opened collecting duct probed with anti-AQP2 (*pseudocolor red*) antibodies to discriminate PCs (highlighted with *yellow arrows*) and ICs (highlighted with *white arrows*). *B*, the averaged time courses of [Ca^2+^]_i_ changes in PCs (*black*) and ICs (*gray*) upon application of Yoda-1 (shown with a *bar* on top). The time points shown in (*A*) are marked as 1 to 3. Number of individual cells is shown. Six different collecting ducts from three different mice were used for analysis. *C*, the summary graph comparing the magnitudes of Yoda-1-mediated [Ca^2+^]_i_ elevations calculated as the difference in [Ca^2+^]_i_ values before (time point 1), at the beginning (time point 2), and at the end (time point 3) of Yoda application in individual PCs and ICs from the conditions in *A*. *Bars* and *whiskers* represent SE and SD, respectively. Mean and median values are denoted with a *dot* and a *line*, respectively. ∗ - significant decreases (*p* < 0.05, one-way ANOVA with post hoc Tukey test) between experimental groups shown with *lines* on the top. AQP2, aquaporine 2.

**Figure 3:**
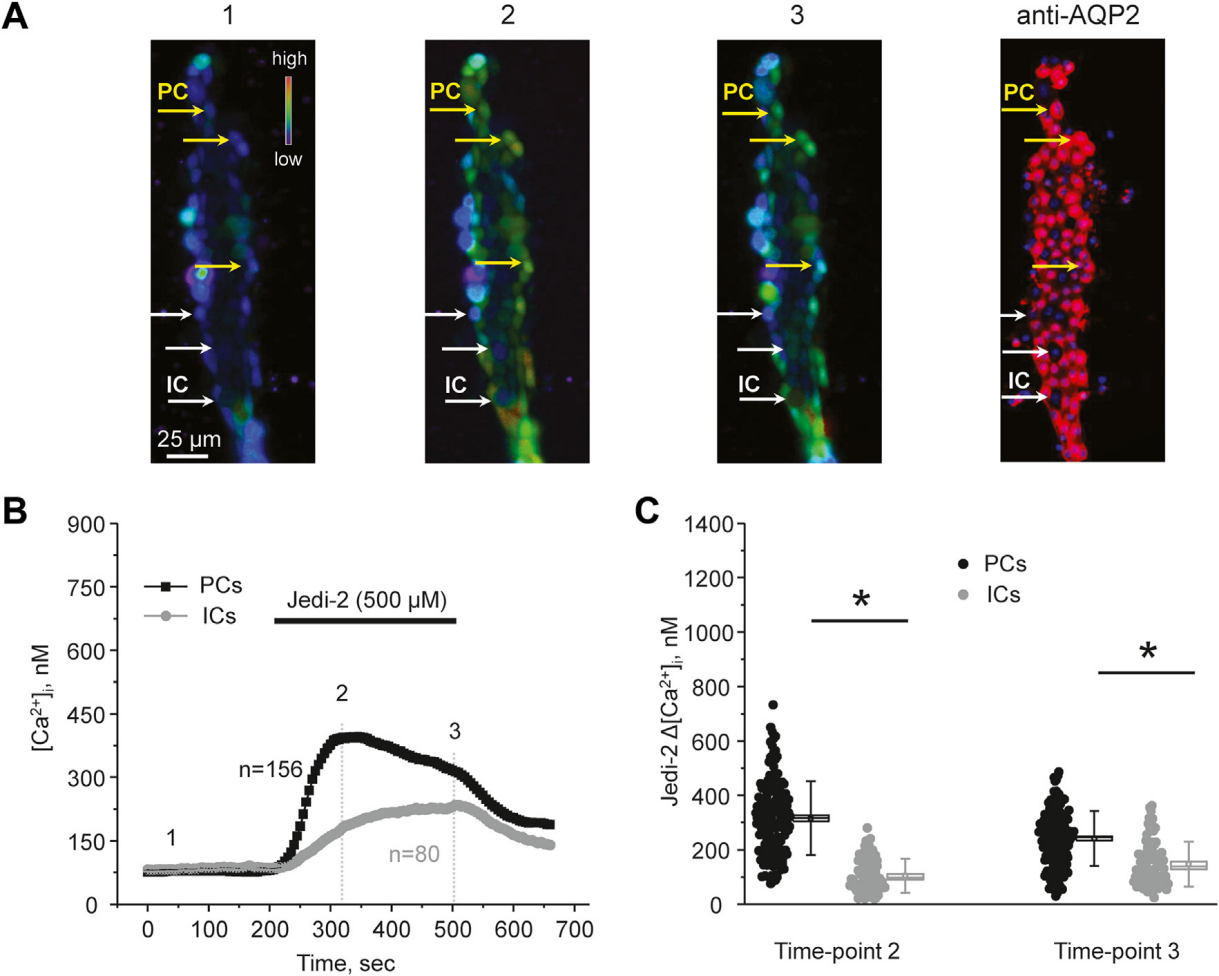
**Stimulation of Piezo1 with Jedi-2 induces greater [Ca**^**2+**^**]**_**i**_**responses in principal (PCs) than in intercalated (ICs) cells of the collecting duct.***A*, representative pseudocolor images (*blue*-low and *red*-high) of an isolated split-opened collecting duct loaded with Ca^2+^-sensitive dye fura-2 at the baseline (1), after 30 s (2), and 5 min (3) of 500 μM Jedi-2 application. Shown on the *right* is a confocal micrograph of the same split-opened collecting duct probed with anti-AQP2 (*pseudocolor red*) antibodies to discriminate PCs (highlighted with *yellow arrows*) and ICs (highlighted with *white arrows*). *B*, the averaged time courses of [Ca^2+^]_i_ changes in PCs (*black*) and ICs (*gray*) upon application of Jedi-2 (shown with a *bar* on top). The time points shown in (*A*) are marked as 1 to 3. Number of individual cells is shown. Six different collecting ducts from three different mice were used for analysis. *C*, the summary graph comparing the magnitudes of Jedi-2-mediated [Ca^2+^]_i_ elevations calculated as the difference in [Ca^2+^]_i_ values before (time point 1), at the beginning (time point 2), and at the end (time point 3) of Jedi-2 application in individual PCs and ICs from the conditions in *A*. *Bars* and *whiskers* represent SE and SD, respectively. Mean and median values are denoted with a *dot* and a *line*, respectively. ∗ - significant decreases (*p* < 0.05, one-way ANOVA with post hoc Tukey test) between experimental groups shown with *lines* on the top. AQP2, aquaporine 2.

We next probed whether activation of Piezo1 contributes to intracellular [Ca^2+^]_i_ signaling in the collecting duct cells. Application of a selective channel agonist, Yoda-1 (20 μM for 5 min), induced a robust increase in [Ca^2+^]_i_ in all cells within split-opened area of a freshly-isolated collecting duct (Fig. 2*A*). Interestingly, we detected two distinct patterns of cellular responses to the treatment with Yoda-1 with the majority of cells responding with a rapid large increase followed by a slow decline in [Ca^2+^]_i_, while the remaining population exhibited a slow progressive rise in [Ca^2+^]_i_. Postexperimental staining of the collecting duct with AQP2 revealed that the observed two populations corresponded to PCs and ICs, respectively (Fig. 2, *A* and *B*). The summary graph in Figure 2*C* shows significantly higher Yoda-1-induced [Ca^2+^]_i_ elevations from the baseline (time-point 1) at the beginning (time-point 2) and at the end of application (time-point 3) in PCs than in ICs. As shown in Figure 3, application of another structurally nonrelated selective Piezo1 agonist, Jedi-2 (500 μM for 5 min) also elicited distinct responses in PCs (larger) and ICs (smaller). This directly implies that the observed differences in [Ca^2+^]_i_ dynamics to Piezo1 stimulation are not attributable to a particular channel agonist.

**Figure 4:**
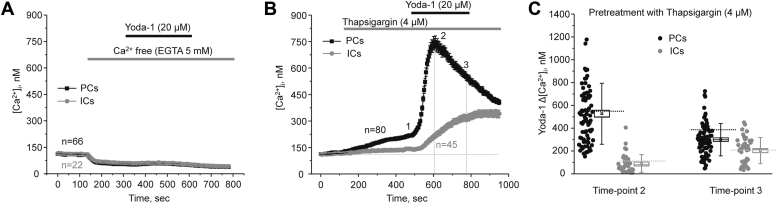
**Direct extracellular Ca**^**2+**^**entry accounts for the Yoda-1 induced [Ca**^**2+**^**]**_**i**_**elevations in the collecting duct.***A*, the averaged time courses of [Ca^2+^]_i_ changes in PCs (*black*) and ICs (*gray*) upon application of Ca^2+^ free medium (5 mM EGTA), followed by a selective Piezo1 agonist Yoda-1 (20 μM). The application times are shown with *gray* and *black bars* on top, respectively. All other conditions are the same as in Figure 2. Number of individual cells is shown. Four different collecting ducts from two different mice were used for analysis. *B*, the averaged time-courses of [Ca^2+^]_i_ changes upon application of the endoplasmic Ca^2+^ ATPase blocker, thapsigargin (4 μM, shown with the *gray bar* on top) followed by Piezo1 agonist, 20 μM Yoda-1 (shown with the *black bar* on top) in individual principal (PCs, *black*) and intercalated (ICs, *gray*) cells within split-opened area of the collecting duct. Number of individual cells is shown. Five different collecting ducts from three different mice were used for analysis. *C*, the summary graph comparing the magnitudes of Yoda-1-mediated [Ca^2+^]_i_ elevations calculated as the difference in [Ca^2+^]_i_ values before (time point 1), at the beginning (time point 2), and at the end (time point 3) of Yoda-1 application in individual PCs and ICs from the conditions in *B*. *Bars* and *whiskers* represent SE and SD, respectively. Mean and median values are denoted with a dot and a line, respectively. *Dashed lines* represent respective average values in control (untreated) conditions.

Yoda-1-induced [Ca^2+^]_i_ elevations might involve both direct Ca^2+^ entry *via* Piezo1 as well as accompanying Ca^2+^ release from the endoplasmic reticulum (ER) intracellular stores. To further test this, removal of extracellular Ca^2+^ (buffered with 5 mM EGTA) abolished responses to Piezo1 stimulation in both PCs and ICs (Fig. 4*A*) suggesting that Yoda-1 triggered a direct Ca^2+^ influx across the plasma membrane. We next quantified responses to Yoda-1 upon inhibition of the endoplasmic Ca^2+^ ATPase with thapsigargin (4 μM) to deplete ER storages. As expected, thapsigargin led to a mild increase in basal [Ca^2+^]_i_ levels, which was more pronounced in PCs than in ICs (Fig. 4*B*). However, this maneuver had no significant effect on the magnitude and different kinetics of Yoda-1-induced [Ca^2+^]_i_ elevations in both PCs and ICs (Fig. 4*C*). This strongly argues against a notable role of the intracellular ER stores in the Ca^2+^ responses to Piezo1 stimulation with Yoda-1. Overall, our results in Figure 2, Figure 3, Figure 4 show that functional Piezo1 is present in all collecting duct cells. However, the Piezo1-dependent Ca^2+^ influx is markedly greater in PCs than in ICs.

### Functional Piezo1 is present at both apical and basolateral membranes of the collecting duct cells

**Figure 5:**
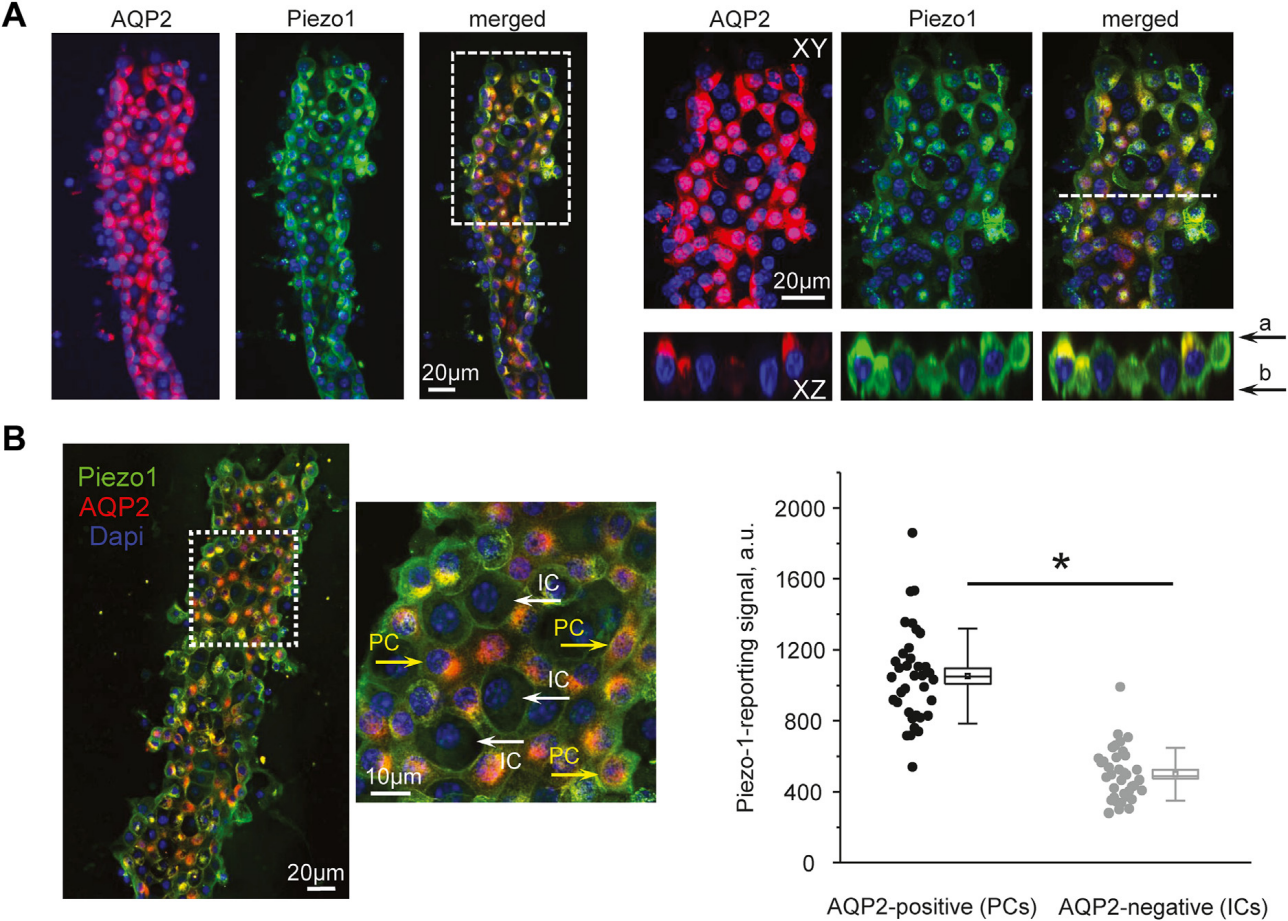
**Piezo1 expression is larger in principal collecting duct cells.***A*, representative confocal images of a split-opened collecting duct probed with anti-AQP2 (*pseudocolor red*, *left panel*), anti-Piezo1 (*pseudocolor green*, *middle panel*) antibodies and the merged image. Nuclear DAPI staining is shown with *pseudocolor blue*. The area depicted with a *white square* is shown on the *right* with a higher magnification. Respective Z-stacks (XZ planes) in the area depicted by the *white dashed lane* are shown below. “a” and “b” denote apical and basolateral sides, respectively. *B*, representative confocal image of a split-opened collecting duct probed with anti-AQP2 (*pseudocolor red*) and anti-Piezo1 (*pseudocolor green*) antibodies similar to that shown in (*A*). The area depicted with a *white square* shows examples of AQP2-positive principal cells (PCs, *yellow arrows*) and AQP2-negative intercalated cells (ICs, *white arrows*). Shown on the *right* is the summary graph of the intensities of Piezo1-reporting signal in PCs and ICs within the same split-opened collecting duct. ∗- significant decrease (*p* < 0.05, one-way ANOVA with post hoc Tukey test) between experimental groups shown with lines on the top. AQP2, aquaporine 2; DAPI, 4′,6-diamidino-2-phenylindole; ICs, intercalated cells; PCs, principal cells.

We next monitored subcellular localization of Piezo1 in split-opened collecting ducts with confocal immunofluorescence microscopy (Fig. 5) to gain insights on the distinct kinetic profiles of [Ca^2+^]_i_ responses to Yoda-1 and Jedi-2 in PCs and ICs. As shown on the representative images of a 3-D scan, Piezo1-reporting signal is distributed on the apical and basolateral sites in all cells (Fig. 5*A*). Importantly, the intensity of the signal was significantly higher in AQP2-positive PCs than in AQP2-negative ICs within the same collecting duct, as summarized in Figure 5*B*. Thus, we show that the lower channel expression is in agreement with lesser [Ca^2+^]_i_ responses to Piezo1 agonists in ICs (Figs. 2 and 4).

**Figure 6:**
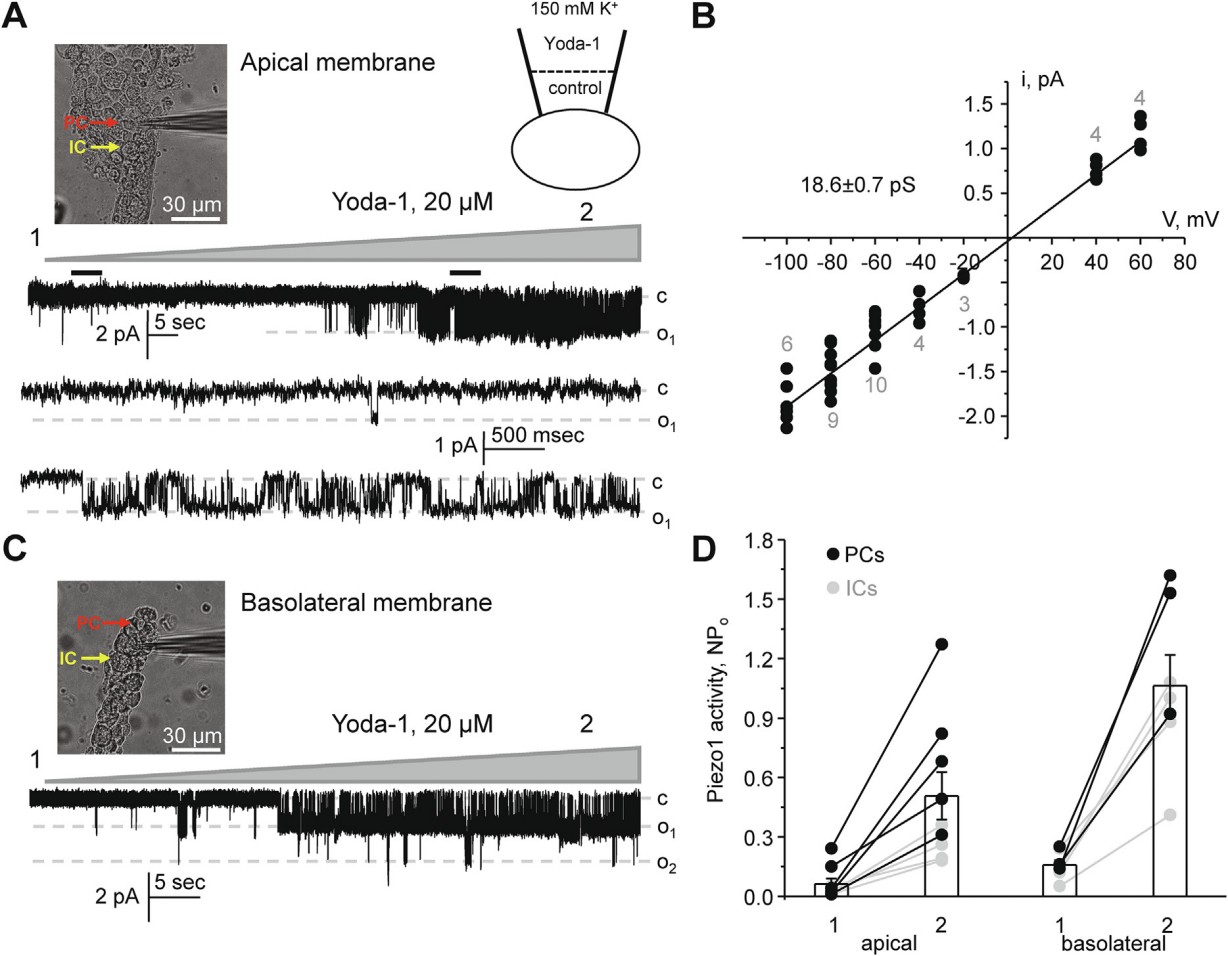
**Piezo1 is functionallyexpressedon both apical and basolateral membranes of the collecting duct cells.***A*, representative continuous current trace from a cell-attached patch clamp experiment on the apical membrane of a cell within a split-opened collecting duct, as shown on the left. Examples of polygonal shape principal (PCs) and round-shape intercalated (ICs) cells are highlighted with *red* and *yellow*, respectively. The pipette was backfilled with Piezo1 agonist Yoda-1 (20 μM), which diffused toward the tip, as shown by a scheme on the top. Inward currents are downward. “c” and “o” denote closed and open states, respectively. The applied pipette voltage was -*V*_p_ = −60 mV. *Black bars* above the trace denote areas shown below with an expanded timescale. *B*, the unitary current (i) – voltage (V) relation of the Yoda-1 activated nonselective cation channel similar to that shown in (*A*). The channel conductance (18.6 ± 0.7 pS) was assessed as a slope of linear fit. The numbers of individual recordings are shown for each applied voltage. *C*, representative continuous current trace from a cell-attached patch clamp experiment on the basolateral membrane of a collecting duct cell, as shown on the left. Examples of polygonal shape principal (PCs) and round-shape intercalated (ICs) cells are highlighted with *red* and *yellow*, respectively. The pipette was backfilled with Piezo1 agonist Yoda-1 (20 μM), which diffused toward the tip, as shown by a scheme on the top. Inward currents are downward. “c” and “o_i_” denote closed and open states, respectively. The applied pipette voltage was -*V*_p_ = −60 mV. *D*, summary graphs comparing changes in Piezo-1 activity (NP_o_) at the start (1) and at the end (2) of Yoda-1 application in cell-attached experiments on the apical and basolateral sides. Experiments on PCs and ICs are highlighted with *black* and *gray* colors, respectively. ICs, intercalated cells; PCs, principal cells.

Since fluorescence Piezo1-reporting signal is apparent at the apical and basolateral sides in the collecting ducts (Fig. 5), we next used patch clamp electrophysiology to inquire whether the channel is indeed functional at both membranes. For this, pipettes were backfilled with Yoda-1 (20 μM) allowing diffusion of the agonist to a patch delineated membrane surface. We observed a gradual activation of a silent or a near silent channel(s) in approximately 30% of cell-attached experiments (10 out of 30) performed on split-opened collecting ducts. A typical experiment is shown in Figure 6*A*. Further analysis revealed that this is a nonselective cation channel with fast kinetics and 18.6 ± 0.7 pS conductance (Fig. 6*B*). These biophysical properties are reminiscent of those reported for Piezo1 in native cells and over-expression systems (4, 7). A Yoda-1-stimulated channel with identical properties was also recorded in cell-attached experiments on the basolateral membrane in freshly isolated collecting ducts (Fig. 6*C*). A summary graph in Figure 6*D* quantifies activation of Piezo1 by Yoda-1 in cell-attached experiments on the apical and basolateral sides. As is clear, the presence of Yoda-1 in a recording pipette exhibited a comparable activation of Piezo1 on both membranes, though the overall activity (NP_o_) was moderately higher on the basolateral side. We also attempted to distinguish PCs and ICs by morphology (polygonal and round shape, respectively (insets in Fig. 6, *A* and *C*), as described previously (32, 33). As highlighted in gray (Fig. 6*D*), Yoda-1 was less potent in ICs than in PCs, which is consistent with lower [Ca^2+^]_i_ responses (Fig. 2) and expression (Fig. 5) in the former.

### Interplay between mechanosensitive Piezo1 and TRPV4 in the collecting duct

**Figure 7:**
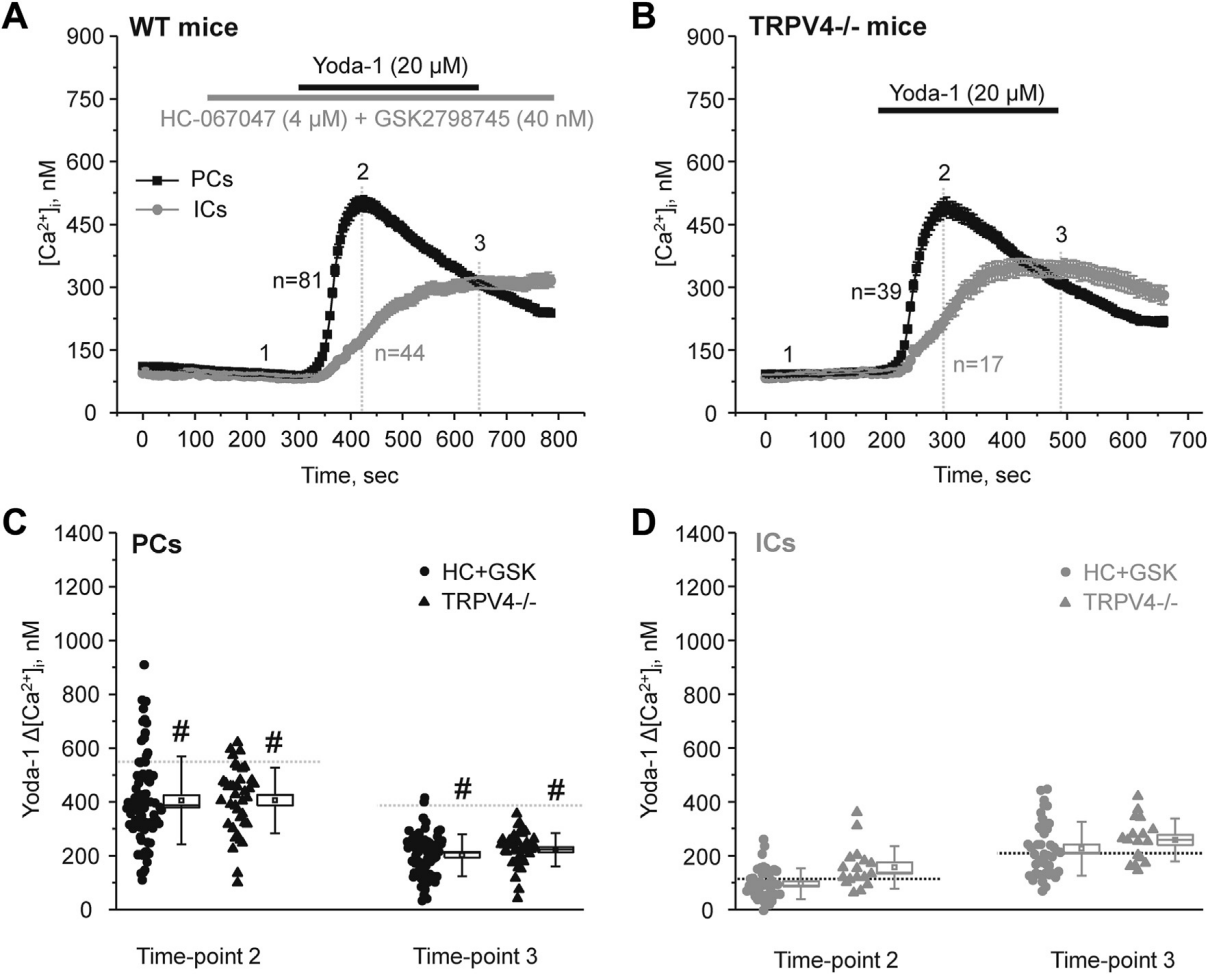
**Moderately decreased Yoda-1-dependent [Ca**^**2+**^**]**_**i**_**responses in the absence of TRPV4.***A*, the averaged time-courses of [Ca^2+^]_i_ changes upon application of selective TRPV4 antagonists HC-067047 (4 μM) and GSK2798745 (40 nM) (shown with the *gray bar* on top) followed by Piezo1 agonist, 20 μM Yoda-1 (shown with the *black bar* on top) in individual principal (PCs, *black*) and intercalated (ICs, *gray*) cells within split-opened area of the collecting duct. Number of individual cells is shown. Five different collecting ducts from three different mice were used for analysis. *B*, the averaged time-courses of [Ca^2+^]_i_ changes upon application of a selective Piezo1 agonist, 20 μM Yoda-1 (shown with the *black bar* on top) in individual principal (PCs, *black*) and intercalated (ICs, *gray*) cells within split-opened area of the collecting ducts isolated from mice with global deletion of TRPV4 (TRPV4−/−). Number of individual cells is shown. Four different collecting ducts from two different mice were used for analysis. The summary graphs comparing the magnitudes of Yoda-1-mediated [Ca^2+^]_i_ elevations calculated as the difference in [Ca^2+^]_i_ values before (time point 1), at the beginning (time point 2), and at the end (time point 3) of Yoda-1 application in individual PCs (*C*) and ICs (*D*) from the conditions in (*A* and *B*). *Bars* and *whiskers* represent SE and SD, respectively. Mean and median values are denoted with a dot and a line, respectively. *Dashed lines* represent respective average values in control (untreated) conditions. # - significant decreases (*p* < 0.05, one-way ANOVA with post hoc Tukey test) *versus* respective control values shown in Figure 2*C*. TRPV4, transitransient receptor potential vanilloid channel type 4.

Piezo1-dependent Ca^2+^ influx was shown to stimulate TRPV4 in different cell types (30, 31). TRPV4 is a mechanosensitive Ca^2+^ permeable channel abundantly expressed in the collecting duct (22, 29). Thus, we next probed potential functional coupling between Piezo1 and TRPV4 in split-opened collecting ducts using pharmacological and genetic tools. In the first set of experiments (Fig. 7*A*), we monitored Yoda-1 stimulation of Piezo1 in PCs and ICs, when TRPV4 activity was blocked by pretreatment with a mixture of selective antagonists HC-067047 (4 μM) and GSK2798745 (40 nM). To exclude an incomplete inhibition and/or putative adverse effects of the blockers, we also documented Piezo1 activation with Yoda-1 in split-opened collecting ducts isolated from TRPV4−/− mice (Fig. 7*B*). In both cases, we observed similar [Ca^2+^]_i_ elevations in PCs (Fig. 7*C*) and ICs (Fig. 7*D*) at the peak (time-point 2) and at the end (time-point 3) of Yoda-1 application. It should be noted that there was a slight decrease in the basal [Ca^2+^]_i_ levels due to the acute TRPV4 inhibition, as we reported previously (34, 35, 36). Thus, the amplitude of Yoda-1-induced [Ca^2+^]_i_ elevations were assessed from this new baseline. Interestingly, inhibition and genetic deletion of TRPV4 led to a moderately (∼25%) but significantly lower responses in PCs when compared to the control values in Figure 2*C*. In contrast, disruption of TRPV4 activity had no measurable effect on Yoda-1-induced [Ca^2+^]_i_ elevations in ICs. Overall, we concluded that stimulation of Piezo1 leads to a partial TRPV4 activation in PCs. The absence of stimulatory effect ICs might be attributed to a much lower Piezo1 expression in this cell type.

### Piezo1 activity in PCs is transiently activated by an increase in fluid flow/tubular stretch

**Figure 8:**
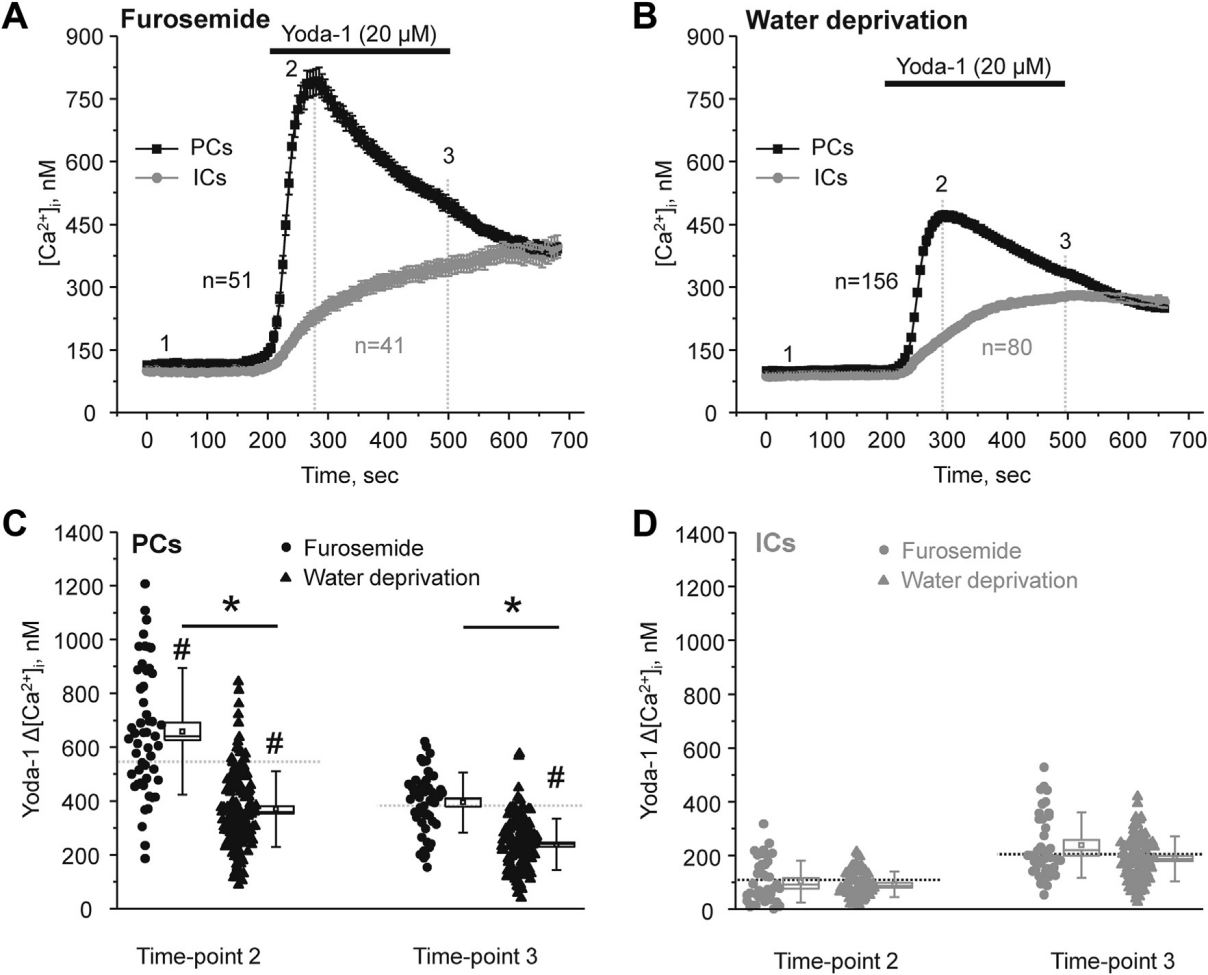
**Short-term changes in flow regulate Piezo1-dependent [Ca**^**2+**^**]**_**i**_**signaling in the collecting duct principal cells.***A*, the averaged time-courses of [Ca^2+^]_i_ changes upon application of a selective Piezo1 agonist, 20 μM Yoda-1 (shown with the *black bar* on top) in individual principal (PCs, *black*) and intercalated (ICs, *gray*) cells within split-opened area of the collecting ducts isolated from mice injected with furosemide (2 mg/kgBW) 18 h and 2 h prior to experiments. Number of individual cells is shown. Four different collecting ducts from two different mice were used for analysis. *B*, the averaged time-courses of [Ca^2+^]_i_ changes upon application of a selective Piezo1 agonist, 20 μM Yoda-1 (shown with the *black bar* on top) in individual principal (PCs, *black*) and intercalated (ICs, *gray*) cells within split-opened area of the collecting ducts isolated from mice subjected to water deprivation for 24 h before experiments. Number of individual cells is shown. Six different collecting ducts from three different mice were used for analysis. The summary graphs comparing the magnitudes of Yoda-1-mediated [Ca^2+^]_i_ elevations calculated as the difference in [Ca^2+^]_i_ values before (time point 1), at the beginning (time point 2), and at the end (time point 3) of Yoda-1 application in individual PCs (*C*) and ICs (*D*) from the conditions in (*A* and *B*). *Bars* and *whiskers* represent SE and SD, respectively. Mean and median values are denoted with a *dot* and a *line*, respectively. *Dashed lines* represent respective average values in control (untreated) conditions. ∗- significant decrease (*p* < 0.05, one-way ANOVA with post hoc Tukey test) between experimental groups shown with lines on the top. # - significant decreases (*p* < 0.05, one-way ANOVA with post hoc Tukey test) *versus* respective control values shown in Figure 2*C*.

Piezo1 was identified as a mechanosensitive channel rapidly activated by a direct physical distortion of the plasma membrane (3, 4). Thus, we next hypothesized that physiological states associated with variations in tubule fluid flow rates affect Piezo1-mediated Ca^2+^ influx in the collecting duct. For this, we first assessed Yoda-1-induced [Ca^2+^]_i_ responses in PCs and ICs from collecting ducts of mice injected with diuretic furosemide (2 mg/kgBW, 18 h and 2 h before the experiment) to increase flow in the collecting duct (Fig. 8*A*). In the second set, experimental animals were water deprived for 24 h to induce antidiuresis (Fig. 8*B*). As it is clear from the averaged time-courses in Figure 8, *A* and *B* and summary graph in Figure 8*C*, pretreatment with furosemide significantly increased, whereas water restriction significantly decreased Yoda-1-dependent [Ca^2+^]_i_ elevations in PCs *versus* untreated control condition. In contrast, we did not detect notable changes in the responses to Piezo1 stimulation with Yoda-1 in ICs under all tested conditions (Fig. 8*D*).

**Figure 9:**
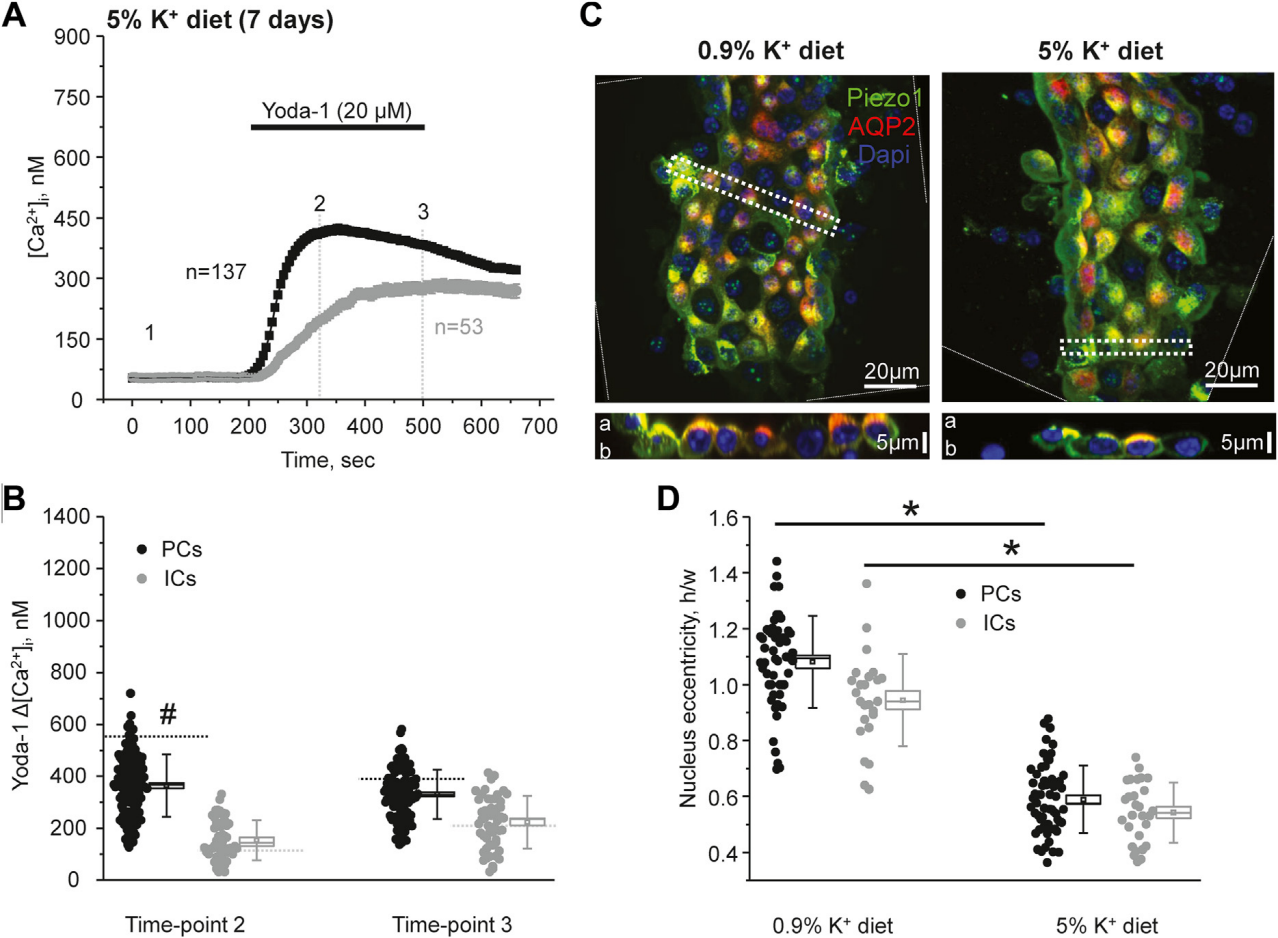
**Long-term stimulation of fluid flow in the collecting duct by high K**^**+**^**diet decreases Piezo1-mediated Ca**^**2+**^**influx in principal cells.***A*, the averaged time-courses of [Ca^2+^]_i_ changes upon application of a selective Piezo1 agonist, 20 μM Yoda-1 (shown with the *black bar* on top) in individual principal (PCs, *black*) and intercalated (ICs, *gray*) cells within split-opened area of the collecting ducts isolated from mice fed a high potassium (5% K^+^) diet for 1 week. Number of individual cells is shown. Six different collecting ducts from three different mice were used for analysis. *B*, the summary graph comparing the magnitudes of Yoda-1-mediated [Ca^2+^]_i_ elevations calculated as the difference in [Ca^2+^]_i_ values before (time point 1), at the beginning (time point 2), and at the end (time point 3) of Yoda-1 application in individual PCs and ICs from the conditions in *A*. *Bars* and *whiskers* represent SE and SD, respectively. Mean and median values are denoted with a *dot* and a *line*, respectively. *Dashed lines* represent respective average values in control (untreated) conditions. # - significant decreases (*p* < 0.05, one-way ANOVA with post hoc Tukey test) *versus* respective control values shown in Figure 2*C*. *C*, representative high magnification confocal images of split-opened collecting ducts probed with anti-aquaporine 2 (*pseudocolor red*) and anti-Piezo-1 (*pseudocolor green*) isolated from mice kept on regular (0.9% K^+^) and high potassium (0.9% K^+^) diets. Nuclear DAPI staining is shown with *pseudocolor blue*. Images were rotated to achieve vertical position of the collecting ducts. Side areas with no data are separated by thin *white dashed lines*. Respective Z-stacks (XZ planes) in the area depicted by the thick *white dashed lane* are shown below. “a” and “b” denote apical and basolateral sides, respectively. *D*, summary graph comparing nuclear eccentricity in principal (PCs) and intercalated cells (ICs) in freshly isolated split-opened collecting ducts from mice kept on regular (0.9% K^+^) and high potassium (5% K^+^) diets. Four different collecting ducts from two different mice were used for analysis. For each individual cell, nuclear eccentricity was assessed as the ratio of the maximal height to maximal widths of DAPI fluorescence. ∗- significant decrease (*p* < 0.05, one-way ANOVA with post hoc Tukey test) between experimental groups shown with lines on the top. DAPI, 4′,6-diamidino-2-phenylindole.

We next investigated the consequences of a continuous elevation in tubular flow in the collecting duct. For this purpose, mice were fed high KCl (5% K^+^) diet for 7 days, which led to at least 3-fold increase in urinary output (28). Unexpectedly, Yoda-1 application induced smaller [Ca^2+^]_i_ responses in PCs (Fig. 9, *A* and *B*), which were comparable to those observed in the state of antidiuresis (Fig. 8*C*). Again, there were no detectable changes in the magnitude of Yoda-1-induced responses in ICs. A similar reduction in Yoda-1-induced [Ca^2+^]_i_ responses in PCs was also observed when Cl^-^ was replaced with organic anions (bicarbonate/citrate) in high K^+^ diet (not shown). Immunofluorescence confocal microscopy studies showed that the intensity of Piezo1-reporting signal (under identical laser settings) was similar in collecting ducts from mice kept on regular and high K^+^ diets (Fig. 9*C*). We also did not detect apparent changes in the subcellular distribution, where Piezo1 was present at the apical and basolateral sides in both tested groups. At the same time, we noted a notable flattening of the collecting duct cells, which could reflect cytoskeleton rearrangement due to prolonged flow-induced shear stress. To exclude potential distortion of cell size during these *ex vivo* preparations, we quantified the changes in nucleus shape and specifically eccentricity (ratio of maximal height to width), which is known to be an index of cytoskeleton rearrangement due to prolonged mechanical stress of the plasma membrane (37). As shown in Figure 9*D*, nuclear eccentricity was significantly decreased during the high K^+^ diet similarly in both PCs and ICs. Since Piezo1 activation by mechanical forces critically depends on the local membrane environment (6), it is reasonable to propose that overstretching of Piezo1-mechanosensitive blades due to cell flattening decreases its sensitivity to respond to mechanical stress.

Overall, the results in Figures 8 and 9 suggest that short-term increases in fluid flow augment Yoda-1-induced [Ca^2+^]_i_ responses in PCs of the collecting duct, whereas sustained elevations in flow rate/perfusion pressure cause rearrangement of cell shape likely rendering Piezo1 inactivation without notable changes in its levels.

### Metabolic acidosis but not metabolic alkalosis increases Piezo1 activity in ICs

**Figure 10:**
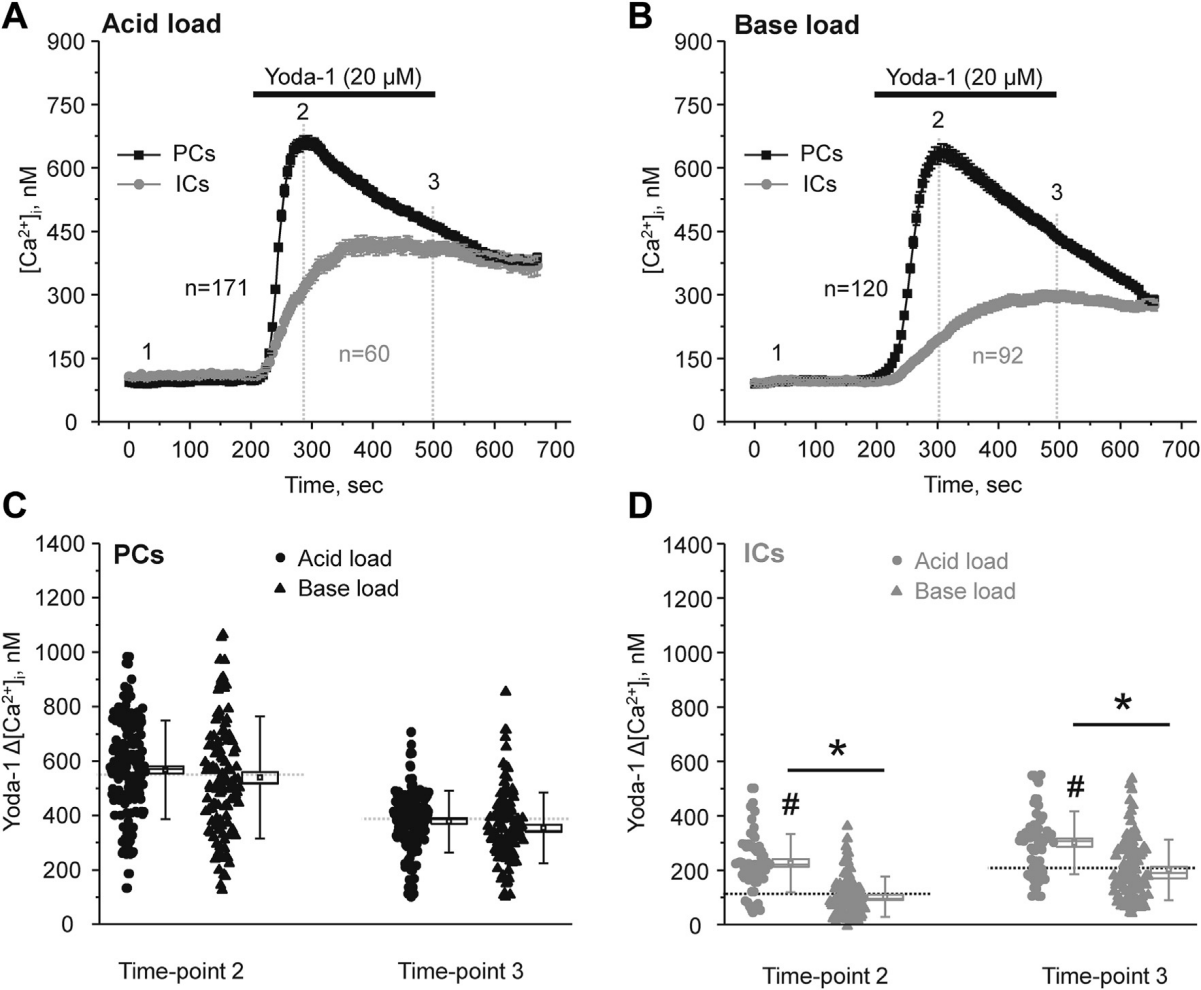
**Metabolic acidosis stimulates Yoda-1-induced [Ca**^**2+**^**]**_**i**_**responses in the collecting duct intercalated cells.***A*, the averaged time-courses of [Ca^2+^]_i_ changes upon application of a selective Piezo1 agonist, 20 μM Yoda-1 (shown with the *black bar* on top) in individual principal (PCs, *black*) and intercalated (ICs, *gray*) cells within split-opened area of the collecting ducts isolated from mice kept on ammonium (NH_4_Cl, 280 mM) water for three days prior to experiments to induce metabolic acidosis (acid load). Number of individual cells is shown. Six different collecting ducts from three different mice were used for analysis. *B*, the averaged time-courses of [Ca^2+^]_i_ changes upon application of a selective Piezo1 agonist, 20 μM Yoda-1 (shown with the *black bar* on top) in individual principal (PCs, *black*) and intercalated (ICs, *gray*) cells within split-opened area of the collecting ducts isolated from mice kept on bicarbonate (NaHCO_3_, 280 mM) water for three days to induce metabolic alkalosis (base load). Number of individual cells is shown. Five different collecting ducts from three different mice were used for analysis. The summary graphs comparing the magnitudes of Yoda-1-mediated [Ca^2+^]_i_ elevations calculated as the difference in [Ca^2+^]_i_ values before (time point 1), at the beginning (time point 2), and at the end (time point 3) of Yoda-1 application in individual PCs (*C*) and ICs (*D*) from the conditions in (*A* and *B*). *Bars* and *whiskers* represent SE and SD, respectively. Mean and median values are denoted with a dot and a line, respectively. *Dashed lines* represent respective average values in control (untreated) conditions. ∗- significant decrease (*p* < 0.05, one-way ANOVA with post hoc Tukey test) between experimental groups shown with lines on the top. # - significant increase (*p* < 0.05, one-way ANOVA with post hoc Tukey test) *versus* respective control values shown in Figure 2*C*. ICs, intercalated cells; PCs, principal cells.

The lack of regulation of Piezo1-dependent [Ca^2+^]_i_ elevations in ICs by variations in fluid flow implies that additional mechanisms might be involved. ICs are known to play a central role in regulation of systemic acid–base balance by secreting H^+^/HCO_3_^-^ to control urinary pH (21, 38). Thus, we next tested whether systemic acid or alkali loads can affect [Ca^2+^]_i_ signaling in response to Piezo1 stimulation in ICs. Mice were kept on NH_4_Cl (280 mM) or NaHCO_3_ (280 mM) water for three days to induce metabolic acidosis or alkalosis, respectively, as described previously (39, 40). Neither treatment had measurable effect on the Yoda-1-induced [Ca^2+^]_i_ responses in PCs, when compared to the control conditions (Fig. 10, *A*–*C*). Importantly, systemic acid load significantly increased Piezo1-dependent [Ca^2+^]_i_ elevations in ICs at both the beginning (time-point 2) and at the end (time-point 3) of Yoda-1 application (Fig. 10*D*). In contrast, no significant changes in Piezo1-dependent Ca^2+^ responses were observed in ICs from mice subjected to base load.

## Discussion

**Figure 11:**
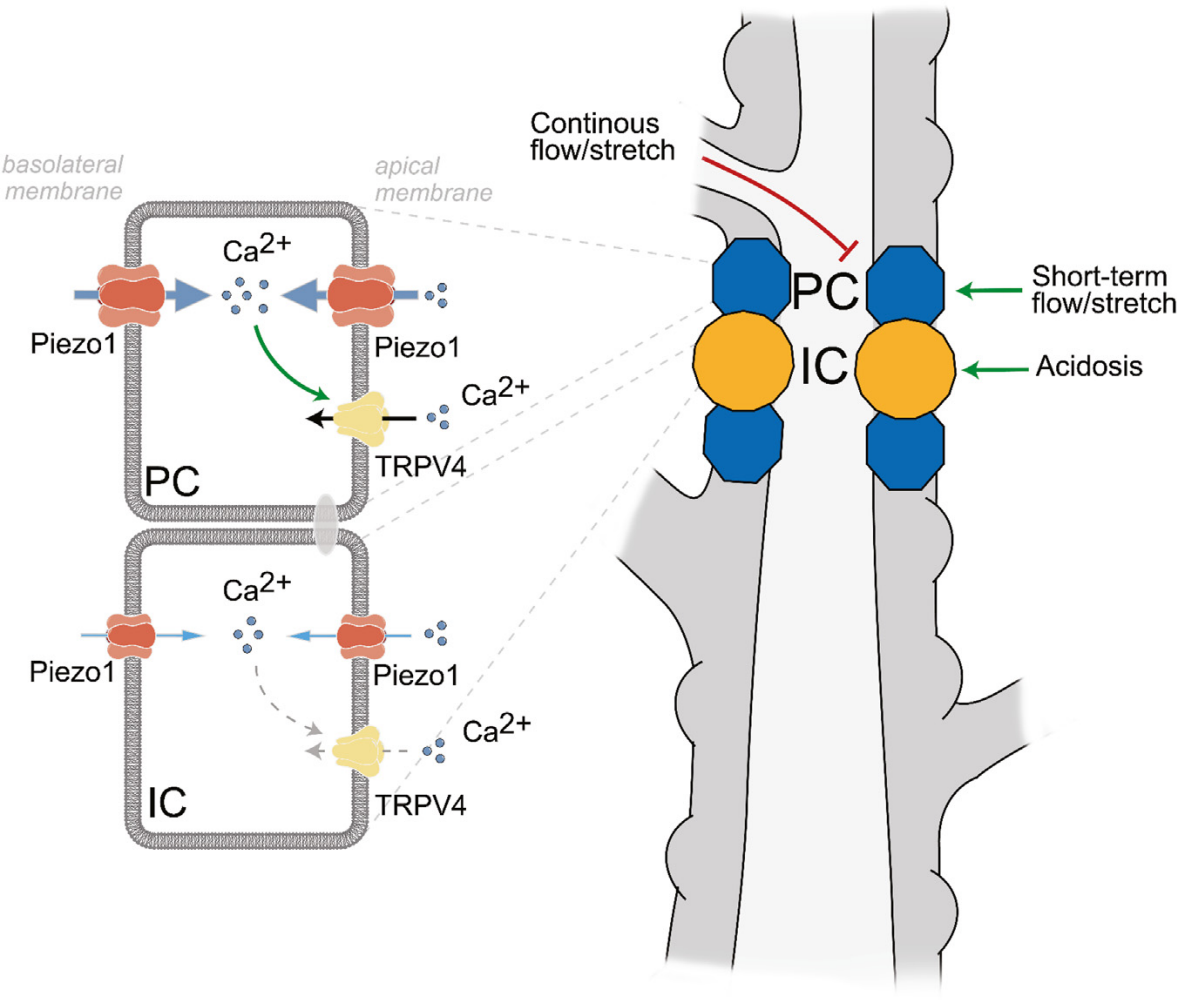
**Principal scheme of Piezo1 function and regulation in principal (PCs) and intercalated (ICs) cells in the collecting duct.** TRPV4–transient receptor potential vanilloid channel type 4. The size of respective icons of Piezo1 and TRPV4 reflects their activity. *Green* and *red arrows* represent stimulatory and inhibitory actions, respectively. TRPV4, transitransient receptor potential vanilloid channel type 4.

In this study, we inquired onto the molecular and signaling determinants of Piezo1 function in the collecting duct cells. Specifically, we showed expression and regulation of the mechanosensitive Piezo1 channel, its interplay with Ca^2+^-permeable TRPV4 in the collecting duct, and revealed cell-specific regulation of [Ca^2+^]_i_ signaling in response to Piezo1 stimulation by systemic cues (Fig. 11). While Piezo1 is present on both apical and basolateral membranes, its expression and Piezo1-dependent Ca^2+^ influx were much greater in PCs than in ICs. Moreover, we also found that the magnitude of Yoda-1-induced [Ca^2+^]_i_ responses positively correlated with acute changes in tubular flow in PCs, whereas Piezo1-dependent [Ca^2+^]_i_ signaling in ICs was augmented by dietary acid load.

Using electrophysiological and imaging techniques, we show that mechanosensitive Piezo1 channel is functional in the collecting duct cells with its activation by Yoda-1 leading to a notable Ca^2+^ influx in both PCs and ICs (Figs. 2 and 6). It has to be noted that quantification of Yoda-1-stimulated [Ca^2+^]_i_ responses offers only an indirect, though convenient, assessment of Piezo1 function. At the same time, we did not detect a significant contribution of intracellular mechanisms, such as Ca^2+^ release from the ER, during pharmacological Piezo1 stimulation with Yoda-1 (Fig. 4). While it seems more accurate to quantify Piezo1 activity in response to mechanical (membrane stretch) rather than pharmacological (with Yoda-1) stimulation, a comparable extension of the plasma membrane attached blade domains has been observed in both cases (6) pointing to a common uniform mode of Piezo1 activation. In addition, there are notable difficulties to accurately measuring Ca^2+^ influx *via* Piezo1 in response to changes in flow-induced shear stress or hypotonicity triggered stretching of the plasma membrane due to rapid channel inactivation in millisecond range. In contrast, application of Yoda-1 allows the assessment of the maximal Piezo1-dependent Ca^2+^ conductance in the collecting duct cells with minor distortions of the accompanying mechanisms, such as Ca^2+^ release from the ER (Fig. 4).

One may think that Piezo1 is ideally positioned to operate as mechanosensor accounting for the commonly observed [Ca^2+^]_i_ elevations in response to changes in flow and osmolarity existing in the collecting duct (25). In contrast, we and others documented that the activity of Ca^2+^-permeable TRPV4 is essential for flow-dependent [Ca^2+^]_i_ elevations in the collecting duct (22, 27, 41). TRPV4 exhibits notable mechanosensitive properties and can be activated by mechanical stress both directly and indirectly, *via* a phospholipase A_2_ (PLA_2_)-dependent signaling cascade (31, 42, 43). Furthermore, increased flow over the apical membrane fails to affect [Ca^2+^]_i_ in the collecting ducts when TRPV4 is deleted or blocked (22, 27, 36). This seeming controversy does not consider rapid inactivation of Piezo1 with channels being already closed within hundreds of milliseconds from the beginning of mechanical distortion of the plasma membrane (4). In contrast to the prolonged opening of the channel by Yoda-1 (Fig. 2), physiologically relevant (*i.e.* mechanical) stimulation of Piezo1 would not lead to sustained mechanosensitive elevations of [Ca^2+^]_i_, which are TRPV4-dependent. Thus, it is reasonable to propose that mechanosensitive Piezo1 and TRPV4 channels have distinct yet complementary functions by tracking the velocity and magnitude of flow changes in the collecting duct, respectively.

An initial transient activation of Piezo1 by mechanical forces was shown to facilitate subsequent TRPV4 channel opening in pancreatic acinar cells with exaggeration of this mechanism leading to the pressure-induced pancreatitis (30). Indeed, TRPV4 is shown to be activated by modest elevations in [Ca^2+^]_i_ *via* a signaling pathway involving calmodulin binding to its C terminus (44). Consistently, we show here that either inhibition or deletion of TRPV4 channel moderately reduced Yoda-1-dependent [Ca^2+^]_i_ elevations in PCs (Fig. 7). This suggests that Piezo1-mediated Ca^2+^ influx is capable of partially stimulating TRPV4. Much lower Piezo1 expression (Fig. 5) and consequently reduced Piezo1-mediated Ca^2+^ influx (Fig. 2) could be responsible for the lack of TRPV4 activation in ICs. Other factors, such as different lipid composition and overall architecture of the plasma membrane of PCs and ICs, could also affect Piezo1 function known to be strongly regulated by local environment (6). It is unlikely, though, that the secondary TRPV4-mediated Ca^2+^ influx could account for the differences in Yoda-1-induced [Ca^2+^]_i_ responses during short-term variations in fluid flow after furosemide and water deprivation (Fig. 8). Thus, we previously showed that sustained elevation of fluid flow in the collecting duct by high K^+^ diet dramatically increased TRPV4 expression and activity (27) to mediate flow-induced K^+^ secretion *via* Ca^2+^-activated big potassium (BK) channels (27, 28). At the same time, we found that this treatment led to a significantly decreased Yoda-1-induced [Ca^2+^]_i_ responses (Fig. 9, *A* and *B*) pointing to reduced Piezo1 activity. While the exact mechanism remains to be further explored, we did not observe changes in Piezo1 expression and subcellular localization in this condition (Fig. 9*C*). Importantly, we detected notable flattening of the collecting duct cells (Fig. 9*D*), suggesting cytoskeleton reorganization due to increased perfusion pressure existing during high K^+^ intake. Published evidence suggest that shear stress mediated signaling to nucleus (monitored as changes in nuclear shape and size) is Ca^2+^-dependent and could be recapitulated by Piezo1 stimulation by Yoda-1 in Madin-Darby canine kidney (MDCK) epithelial cells (45). It is possible that such notable cytoskeleton reorganization due to Piezo1-or potentially TRPV4-dependent Ca^2+^ signaling would lead to reciprocal negative feed-back inhibition of Peizo1 function to limit further structural changes. Alternatively, direct monitoring conformational dynamics of the membrane bound mechanosensitive elements (*i.e.* blades) of Piezo1 using nanoscopic fluorescence imaging revealed that the magnitude of blade expansion correlates with channel activation (6). Thus, it is possible that prolonged shear stress induces deformation of the plasma membrane and overstretching of the blades thereby impeding Piezo1 mechanosensitivity.

The finding that Piezo1 expression (Fig. 3) and activity (Figs. 2 and 5) are high in PCs but low in ICs is intriguing. In fact, ICs are generally larger in size than PCs, thus protruding to the tubular lumen. One may think that they are better suited to sense flow- and tonicity-induced mechanical pressure. However, this view is not supported by the predominant Piezo1 expression in PCs shown in this study. The same pattern was also reported by other groups using Piezo1-reporter transgenic mouse models (17, 19). Moreover, mice with a conditional KO of Piezo1 in the renal tubule have normal urinary concentrating ability but exhibit a delayed urinary dilution after rehydration (17). Since PCs (and not ICs) mediate AQP2-dependent water reabsorption to control urinary osmolarity, the overall phenotype in the KOs is indicative of predominant Piezo1 expression in this cell type. Consistently, we found that Yoda-1-induced [Ca^2+^]_i_ responses in PCs but not ICs are diminished during water deprivation but are augmented by an acute increase in flow due to furosemide (Fig. 8). It is worth noting that the expression of mechanosensitive TRPV4 channel is also higher in PCs, though the difference is not that large (22). Interestingly, expression of mechanosensitive volume-regulated anion channels (VRACs, also known as SWELLs) and particularly LRRC8A/E heteromer has been specifically reported in ICs but not PCs (46). Such a striking separation of the cation- and anion-selective mechanosensitive channels to PCs and ICs, respectively is reminiscent of the predominant cation-selective (mostly K_ir_4.1/5.1 potassium channel) and anion-selective (ClC-K2 chloride channel) basolateral conductance in these cell types (47, 48, 49). It is plausible to propose that such separation of cationic and anionic permeability is instrumental for independent physiological roles of PCs and ICs requiring different mechanisms of mechanosensitivity in fine-tuning water and electrolyte transport in the collecting duct.

Our patch clamp data (Fig. 6) provide a direct evidence of the presence of a ∼19 pS Yoda-1 activated channel on both apical and basolateral membranes of the collecting duct cells. The observed biophysical profile is very similar to that reported for Piezo1 (4, 7). The overall channel activity (NP_o_) after stimulation by Yoda-1 seems to be greater in polygonal-shape PCs than in round-shape ICs and on the basolateral side more than on the apical side. These results are in a good agreement with immunofluorescence detection of Piezo1 in renal sections (Fig. 1) and isolated split-opened collecting ducts (Fig. 5). Consistently, Piezo1-reporting transgenic mouse models also found the most abundant signal on the basolateral side of PCs in the collecting duct (17, 19). It is currently not established whether Piezo1 has different functional roles on the apical *versus* basolateral membranes. However, it might be reasonable to assume that the apical Piezo1 is better suited to sense acute changes in fluid flow and to facilitate TRPV4 activation (which is preferentially on that side (22)), whereas basolateral Piezo1 could be instrumental in sensing the overall pressure/tension arising from either osmotic gradients or substantial increases in fluid delivery to the collecting duct. The lower Piezo1 expression in ICs indicates that the channel is not likely playing a major role in acid–base transport at this site. On the other hand, we observed a specific upregulation of Piezo1 in ICs in response to metabolic acidosis (Fig. 9). Future studies are necessary to examine the potential contribution of Piezo1 for renal adaptation to systemic acid–base stimuli.

## Experimental procedures

### Reagents

All chemicals and materials were from Sigma-Aldrich, VWR, Thermo Fisher Scientific, and Tocris Bioscience unless noted otherwise and were at least of reagent grade.

### Research animals

Animal use and welfare adhered to the NIH Guide for the Care and Use of Laboratory Animals following protocols reviewed and approved by the Animal Care and Use Committee of the University of Texas Health Science Center at Houston. For experiments, male C57BL/6J (Charles River Laboratories) and TRPV4−/− (22, 50) mice 6 to 10 weeks old were used. Animals were maintained on standard rodent chow and had free access to tap water unless specified otherwise. To increase urinary output, mice were fed high KCl diet (5% K^+^, TD150699), and high KBicarbonate/citrate (KB/C) diet (5% K^+^ with bicarbonate to citrate in 4:1 ratio, TD150759) for 1 week. All diets were purchased from Envigo. Diuretic furosemide (2 mg/kgBW; TCI America; Cat. # F01825G) was injected intraperitoneally 18 h and 2 h prior to experimentation. To induce metabolic acidosis/alkalosis, animals were given water supplemented with 280 mM NH_4_Cl + 0.5% sucrose (acid load) or 280 mM NaHCO_3_ + 0.5% sucrose (base load) for three days, respectively, as was described previously (39, 40).

### Isolation and split-opening of cortical collecting ducts

The procedure for isolation of the cortical collecting ducts from mouse kidneys suitable for fluorescence [Ca^2+^]_i_ measurements and patch clamp electrophysiology closely followed the protocols previously published by our group (39, 51). Kidneys were cut into thin slices (<1 mm) with slices placed into an ice-cold bath solution containing (in mM): 150 NaCl, 5 KCl, 1 CaCl_2_, 2 MgCl_2_, 5 glucose, and 10 Hepes (pH 7.35). Straight cortical-to-medullary sectors, containing approximately 30 to 50 renal tubules, were isolated by microdissection using watchmaker forceps under a stereomicroscope. To dissolve the basal lamina and to get direct access to the basolateral membrane, isolated sectors were further incubated in the Ringer’s solution containing 0.8 mg/ml collagenase type I (Alfa Aesar) and 5 mg/ml of dispase II (Roche Diagnostics) for 12 min at 37 °C followed by extensive washout. Cortical collecting ducts were visually identified by their morphological features (pale color; coarse surface) and by their postexperimental staining with anti-AQP2 (Fig. 2 for example). The collecting ducts were mechanically isolated from kidney slices by microdissection using watchmaker forceps under a stereomicroscope. Isolated collecting ducts were attached to 5 × 5 mm cover glasses coated with poly-L-lysine. A cover-glass containing a collecting duct was placed in a perfusion chamber mounted on an inverted Nikon Eclipse Ti-S microscope and perfused with bath solution at room temperature. Cortical collecting ducts were further split opened with two sharpened micropipettes, controlled with different micromanipulators, to reliably monitor [Ca^2+^]_i_ signals from individual cells. The collecting ducts were used within 2 h of isolation.

### Intracellular Ca^2+^ measurements

Split-opened cortical collecting ducts were loaded with fura-2 by incubation with 2 μM fura-2/AM acetoxymethyl ester in the bath solution for 40 min at room temperature followed by a washout with the bath solution for additional 10 min. Collecting ducts were placed in an open-top imaging study chamber (RC-26GLP; Warner Instruments) with a bottom coverslip viewing window and the chamber attached to the microscope stage of a Nikon Ti-S Wide-Field Fluorescence Imaging System (Nikon Instruments) integrated with Lambda XL light source (Sutter Instrument) and QIClick 1.4 megapixel monochrome CCD camera (QImaging) *via* NIS Elements 4.3 Imaging Software (Nikon Instruments). Cells were imaged with a 40× Nikon Super Fluor objective and regions of interest were drawn for individual cells. For [Ca^2+^]_i_ measurements, the fura-2 fluorescence intensity ratio was determined by excitation at 340 nm and 380 nm and calculating the ratio of the emission intensities at 511 nm in the usual manner every 5 s. The changes in the ratio were converted into changes in intracellular calcium, as we described in great details previously (52). Minor fura-2 bleaching during the timeline of experiments was corrected. Experiments were performed under permanent perfusion of a solution containing (in mM): 150 NaCl, 5 KCl, 1 CaCl_2_, 2 MgCl_2_, 5 glucose, and 10 Hepes at 1.5 ml/min rate. Ca^2+^-free medium was prepared from the bath solution by removal of CaCl_2_ and addition of 5 mM EGTA. In average, six individual collecting ducts (30–50 cells in each) from three mice were used for each experimental set.

### Immunofluorescence microscopy (split-opened collecting ducts)

Following [Ca^2+^]_i_ measurements, split-opened collecting ducts were fixed with 10% neutral buffered formalin (Azer Scientific; Cat. # PFNBF240) for 15 min at room temperature. After fixation, the samples were permeabilized by addition of 1% SDS (Sigma-Aldrich, Cat. # L4390) in PBS for 10 min and washed in PBS two times for 10 min. Nonspecific staining was blocked with 10% normal goat serum (Novus Biologicals; Cat. # NBP223475) in PBS for 1 h at room temperature. The samples were incubated overnight at +4 °C in dark with rabbit anti-mouse anti-AQP2 antibodies (1:4000, Alomone Labs; Cat. # AQP2-002), washed with PBS, and incubated with goat anti-rabbit Alexa 594 F(ab’) secondary antibodies (1:1000 Jackson ImmunoResearch, Cat. # 111–587–003) for 60 min at room temperature. For double staining protocol, collecting ducts were incubated overnight at +4 °C with conjugated rabbit anti-mouse anti-AQP2-ATTO Fluor-550 (1:200, Alomone Labs; Cat. # AQP2-002-AO) and anti-Piezo1 (1:100, Thermo Fisher Scientific, Cat. # PA577617) antibodies. Followed by washing with PBS for 20 min at room temperature, the samples were incubated with goat anti-rabbit Alexa 647 (H + L) secondary antibodies (1:1500, Thermo Fisher Scientific; Cat. # A32733) for 60 min at room temperature. After washing with PBS (2 times for 5 min) the samples were stained with 4′,6-diamidino-2-phenylindole (DAPI) (300 nM concentration, MilliporeSigma, Calbiochem; Cat. # 5.08741.0001) to visualize nuclei. The samples were mounted with ProLong Gold antifade reagent (Thermo Fisher Scientific; Cat. #P10144). The samples were imaged with a Nikon A1R confocal microscope using 405, 640, and/or 561 nm laser diodes and emission captured with a 16 bit Cool SNAP HQ2 camera (Photometrics) interfaced to a PC running NIS elements software.

### Immunofluorescence microscopy (kidney sections)

Freshly isolated kidneys were decapsulated and placed into cryo tubes with embedding medium (Andwin Scientific Tissue-Tek CRYO-OCT Compound 4583; Cat. # 14-373-65). The kidneys were fixed in 10% neutral buffered formalin at +4 °C overnight and soaked in 0.9 M sucrose overnight at +4 °C. The kidneys were next flash-frozen in liquid nitrogen for 3 to 5 min. Transverse cut 6 μm thick sections were made on CM 1850 cryostat (Leica). The sections were allowed to warm to room temperature, washed with PBS and permeabilized with 0.1% Triton x100 (Sigma-Aldrich; Cat. # 56H0850) for 10 min. After extensive washout, the samples were treated with 10% normal goat serum for an hour at room temperature. Sections were incubated overnight at +4 °C with rabbit anti-mouse anti-Piezo1 (1:50, Thermo Fisher Scientific; Cat. # PA577617) antibodies. Followed by washing with PBS for 20 min at room temperature, the samples were incubated with goat anti-rabbit Alexa 647 (H + L) secondary antibodies (1:1500, Thermo Fisher Scientific; Cat. # A32733) for 60 min at room temperature. Followed by washing with PBS for 20 min at room temperature, the samples were incubated with 5% normal rabbit serum for 30 min at room temperature and further with rabbit anti-mouse anti-AQP2-ATTO Fluor-550 (1:200, Alomone Labs; Cat. # AQP2-002-AO) overnight at +4 °C. After washing with PBS for 20 min at room temperature, nuclei were stained with DAPI (0.5 μg/ml) for 10 min. The samples were mounted with ProLong Gold antifade reagent (Thermo Fisher Scientific; Cat. #P10144). The labeled kidney sections were imaged with a Nikon A1R confocal microscope, as we did similarly before (39). In brief, samples were excited with 405, 640, and/or 561 nm laser diodes and emission captured with a 16 bit Cool SNAP HQ2 camera (Photometrics) interfaced to a PC running NIS elements software.

### Patch clamp electrophysiology

Single-channel activity of Yoda-1-activated Piezo1 channel was determined in cell-attached patches on the apical membrane in freshly isolated split-opened collecting ducts and on the basolateral membrane in freshly isolated collecting ducts, as shown in representative images in Figure 6. Gradual activation of Piezo1 by Yoda-1 was detected under voltage-clamp conditions (−*Vp* =− 60 mV), as previously described (47, 53). To assess single channel conductance, pipette voltage was changed from -Vp = −100 mV to +60 mV for at 30 s. PCs and ICs were visually identified by their polygonal and round shape, respectively. Recording pipettes had resistances of 4 to 8 megaohm. Bath solution was (in mM): 150 NaCl, 5 KCl, 1 CaCl_2_, 2 MgCl_2_, 5 glucose, and 10 Hepes (pH 7.35). Tip of the recording pipette (approximately 3 mm) was filled with control solution containing 150 KCl, 2 MgCl_2_, 10 Hepes, and 5 glucose (pH 7.35). The rest of pipette was filled by the same solution also containing Yoda-1 (20 μM). Gap-free single channel current data from gigaohm seals were acquired and analyzed with Axopatch 200B (Molecular Devices) patch clamp amplifier interfaced *via* a Digidata 1440 (Molecular Devices) to a PC running the pClamp 10.7 suite of software (Molecular Devices). Currents were low-pass filtered at 1 kHz with an eight-pole Bessel filter (Warner Instruments). Events were inspected visually prior to acceptance. Channel activity in individual patches, defined as *NP*_o_, was calculated using the following equation: *NP*_o_ = (*t*_1_ + 2*t*_2_ + … + *nt*_*n*_), where *N* and *P*_o_ are the number of Piezo1 in a patch and the mean open probability of these channels, respectively, and *t*_*n*_ is the fractional open time spent at each of the observed current levels. The stimulatory effect of Yoda-1 was quantified as a difference in NP_o_ (over a 30 s span) at the beginning (control) and after 2 to 3 min, allowing for the Yoda-1 diffusion to the pipette tip.

### Data analysis

All summarized data are reported as mean ± SD (whiskers) and SE (bars). Statistical comparisons were made using one-way ANOVA with post hoc Tukey test or one-way repeated measures ANOVA with post hoc Bonferroni test. *p* value less than 0.05 was considered significant.

## Data availability

All research data are contained within the manuscript. Individual immunofluorescence images and patch clamp recordings could be obtained from the corresponding author upon a reasonable request.

## Conflict of interest

The authors declare that they have no conflicts of interest with the contents of this article.
